# Outcomes of Incidental Durotomy Repair in Thoracolumbar Spine Surgery: An Institutional Experience With Orthopedic Residents

**DOI:** 10.7759/cureus.41740

**Published:** 2023-07-11

**Authors:** Arun Kumaar, Manoj K Ramachandraiah, Sandesh Agarawal, Arun H Shanthappa, Madhavan Parmanantham

**Affiliations:** 1 Orthopedics, Sri Devaraj Urs Medical College, Kolar, IND; 2 Orthopedics, Sri Devaraj Urs Academy of Higher Education and Research, Kolar, IND

**Keywords:** spine surgery, thoracolumbar spine surgery, thoracolumbar spine, visual analog scale, oswestry disability index, durotomy

## Abstract

Background

The occurrence of incidental durotomies (IDs) following spinal operations is a widely recognized issue. Complications such as poor outcomes, extended hospitalization, prolonged immobilization, infections, and revision surgeries are all potential consequences of inadequate durotomy management during the initial surgery. This study aims to describe the outcomes of ID repair in thoracolumbar spine surgery in terms of the Oswestry Disability Index (ODI) score and visual analog scale (VAS) when performed with the active involvement of orthopedic residents in the surgical procedure.

Methodology

Between April 2021 and April 2023, a hospital-based observational study was conducted among 110 patients hospitalized in the orthopedic ward at R.L. Jalappa Hospital and Research Center in Kolar, Karnataka, who required IDs due to an accidental dural tear or a postoperative CSF fluid leak following thoracolumbar spine procedures. Patients with a previous history of thoracolumbar spine surgery, vertebral tumors, spinal metastasis, infections, e.g., spondylodiscitis, or Pott's spine were excluded. The ODI score and VAS score were calculated on the postoperative day, one month, and three months following surgery.

Results

The mean age of the study participants was 62.81 + 10.49 years, with a male preponderance of 67.2% among the study participants. The mean BMI of study participants was 23.77 kg/m2. Approximately 24.5% of participants had a prior history of spinal surgery. Among 110 patients, 32 had postoperative complications. Six patients reported experiencing urinary retention, followed by five with CSF leakage and one with a postural headache (five cases). Based on the ODI score, mild disability was seen in 32.7% of the study samples at three months of follow-up. Based on the VAS score, moderate pain was seen among all the study samples at three months of follow-up. The ANOVA test revealed statistically significant differences in ODI and VAS score reductions between the immediate postoperative period and the one-month and three-month follow-up periods (p = 0.001 and p = 0.0247, respectively).

Conclusion

Less than one-third of the samples had postoperative complications. At three months, ODI scores showed mild disability in one-third of the study samples. At three months, all study samples had moderate VAS pain. The improvement in ODI and VAS scores from the day after surgery through the one-month and three-month follow-up periods was statistically significant.

## Introduction

Incidental durotomy (ID), also known as spinal puncture, is a common complication of spinal surgery that is caused by an accidental puncture of the dural sheath of the spine during the operative procedure [1]. Globally, the prevalence of dural tears ranges from one to 17, with a baseline prevalence of approximately 3.5% [2,3].

Incidental dural tears are unavoidable even in the hands of experienced spine surgeons; nonetheless, careful treatment is essential to avoid problems. The risk of ID is attributed to a variety of factors, including advanced age, gender, a prior history of spinal surgery, a high body mass index, patients who have undergone either laminectomy, osteotomy, or interbody procedures, as well as high-risk anatomical areas, including the caudal margins of the cranial lamina and herniated discs, as well as the medial aspect of the facet joints [4].

The adverse effects of a mismanaged durotomy in the initial procedure include poor outcomes, extended hospitalization, prolonged immobilization, infection, and revision surgery. Additionally, minor postoperative complications may occur, such as headaches, nausea, vomiting, and neck pain. Furthermore, back pain and dizziness may occur, as well as diplopia and photophobia. Tinnitus and blurred vision may also occur [5,6]. These symptoms are due to a persistent leakage of cerebrospinal fluid (CSF) from the subarachnoid space. As a result, the pressure of this fluid decreases, resulting in a decrease in buoyancy and the displacement of intracranial contents through the caudal region [7].

There is no standardized approach for the repair of large and pinhole-shaped durotomies. The primary repair, application of fibrin adhesive, use of fleeces with fibrin coating, and use of muscle grafts all vary from surgeon to surgeon [8]. Generally, surgeons who are trained in the field of spine surgery are responsible for the operation, so the risk of an accidental dural tear is minimal.

The acquisition and advancement of knowledge are essential components of any surgeon's education; however, the volume of cases and the number of years of experience are the two primary determinants of surgical performance [9,10]. Surgeon experience has been extensively discussed in the context of three-column backbone osteotomies, where years of experience have been shown to be the primary determinant of adverse outcomes [11]. However, there is no peer-reviewed literature comparing the experience of a surgeon with the results of an inadvertent durotomy repair performed in thoracolumbar spine surgery and its management. Although there is a large amount of data available, there is a great deal of disagreement amongst spine surgeons regarding the best approach for intra- and postoperative CSF leaks, particularly due to the lack of evidence-based guidelines.

So, the current study aimed to investigate the outcomes of ID repair in thoracolumbar spine surgery with orthopedic residents. The clinical results were assessed in terms of the Oswestry Disability Index (ODI) score and the visual analog scale (VAS) during the initial postoperative period (immediate postoperative), one-month, and three-month postoperative periods.

## Materials and methods

Study design and settings

This hospital-based observational study included 110 patients who had experienced an accidental dural tear or a postoperative CSF leak following thoracolumbar spine surgeries and presented to the Department of Orthopedics, R.L. Jalappa Hospital and Research Center in Kolar, Karnataka, during the period from April 2021 to April 2023. The operations were performed by one spine fellowship-trained orthopedic surgeon with senior residents as first and second assistants who are actively involved in critical surgical steps of neural decompression.

Ethics clearance

Data collection was performed after obtaining ethical approval from the Institutional Ethics Committee of Sri Devaraj Urs Medical College, Kolar, Karnataka, India (approval number: DMC/KLR/IEC/171/2022-23).

Selection criteria

Patients of all age groups and genders who underwent thoracolumbar spine surgery were included in the study. The exclusion criteria were patients with a previous history of thoracolumbar spine surgery, vertebral tumors, spinal metastasis, infection, e.g., spondylodiscitis, and Pott's spine.

Sample size and sampling method

A retrospective review of 2144 patients by Cammisa et al. in 2000 noticed that the incidence of accidental durotomy was 3.1% [12]. Considering this incidence, the minimum sample of 70 was calculated using the formula of 3.84*pq/d^2^, where prevalence (p) = 3.1, q (1-p) = 96.9, and precision (d) = 0.04, with a 95% confidence interval. During the time period from April 2021 to April 2023, all consecutive patients who had suffered an accidental dural tear or a postoperative CSF leak following thoracolumbar spine surgeries presented to the department.

Data collection procedure

The data were collected using electronic case records, which contain socio-demographic and clinical histories, as well as outcome information for the patients. Urinary retention, postural headache, CSF leakage, deep vein thrombosis, wound dehiscence, hematoma, and other complications were taken into account. The ODI score and the VAS were calculated on the postoperative day, one month, and three months after surgery.

When it comes to measuring the effectiveness of hospital care for low back pain, the ODI is the gold standard [13,14]. It is a 10-section, self-administered questionnaire made to evaluate limitations in different daily activities on a scale of 0 to 5, with 5 denoting the greatest disability, and each section is graded.

The VAS is a reliable, self-reported method of gauging both short- and long-term pain [15]. Marks are made by hand on a 10-cm line that represents a scale from "no pain" to "worst pain," with each endpoint being recorded.

Data analysis

The data were entered into an Excel spreadsheet in Microsoft Office (Microsoft Corporation, Redmond, WA) and analyzed with SPSS version 21 (IBM Corp., Armonk, NY). The quantitative variable data were presented as the mean and standard deviation. The qualitative information was presented as a frequency and percentage distribution. The analysis of variance (ANOVA) test was used to compare the mean difference between the OWI score and VAS in the immediate postoperative period, as well as the one-month and three-month follow-up periods. For statistical significance, the p-value was considered below 0.05.

## Results

The mean age of the study participants was 62.81 ± 10.49 years. A total of 38.2% of cases belonged to the 61-70 age group, while 22.7% belonged to the 51-60 age group. Only nine cases were older than 80 years. Out of the total patients, 74 (67.3%) were males and 36 (32.7%) were females. The preponderance of males was higher than females. The mean BMI of the study participants was 23.77 ± 2.26 kg/m^2^. The mean ICU stay of patients was 7.3 ± 3.3 days after the operative procedure.

Among the patients, 15.5% had the presence of hypertension, and 14.5% had diabetes mellitus. Overall, 16.4% of cases had a history of cigarette smoking, 8.2% had a history of tobacco chewing, and 8.2% had a history of alcoholic beverage consumption. Table 1 shows that 27 of the total cases had a history of spinal surgery.

**Table 1:** Basic characteristics of the study population * Mean ± standard deviation.

Variable		Frequency	Percent
Age in years	<50	20	18.2
	51-60	25	22.7
	61-70	42	38.2
	71-80	14	12.7
	>80	9	8.2
Gender	Male	74	67.3
	Female	36	32.7
BMI		23.77 ± 2.26*	
Intensive care unit stay (in days)		7.3 ± 3.3*	
Comorbidities	Hypertension	17	15.5
	Diabetes mellitus	16	14.5
	None	77	70.0
Addiction history	Smoking	18	16.4
	Tobacco chewing	9	8.2
	Alcohol	9	8.2
	None	74	67.3
History of spinal surgery	Yes	27	24.5
	No	83	75.5

Six patients reported experiencing urinary retention, followed by five with CSF leakage and one with a postural headache (five cases). Myocardial infarction had occurred in one case, and a pulmonary embolism in another. According to Table 2, there were two cases of surgical site infection.

**Table 2:** Postoperative complications among the study participants

Postoperative complications	Frequency	Percent
Wound dehiscence	3	9.4
Urinary retention	6	18.8
Surgical site infection	2	6.3
Pulmonary embolism	1	3.1
Postural headache	5	15.6
Pneumothorax	3	9.4
Myocardial infarction	1	3.1
Hematoma	2	6.3
Deep vein thrombosis	3	9.4
Cerebrospinal fluid leakage	5	15.6
Acute renal failure	1	3.1
Total	32	100.0

Of the people who took part in the study, 89% experienced moderate disability during the postoperative period. Participants in the study reported mild disability after the first postoperative month (60.9%). Table 3 shows that 62.8% of cases showed no disability after three months of follow-up.

**Table 3:** Distribution of ODI score among the study participants (n = 110) ODI: Oswestry Disability Index.

ODI score	Frequency (%)		
	At postoperative period	After 1 month	After 3 months
0-4 (no disability)	0 (0)	12 (10.9)	69 (62.8)
5-14 (mild disability)	21 (19.1)	67 (60.9)	41 (37.2)
15-24 (moderate disability)	89 (80.9)	31 (28.2)	0 (0)
25-34 (severe disability)	0 (0)	0 (0)	0 (0)
35-50 (complete disability)	0 (0)	0 (0)	0 (0)

The majority of study participants (97.6%) experienced severe postoperative pain. After one month of postoperative recovery, 66.4% of study participants reported moderate pain. As shown in Table 4, all patients experienced moderate pain at the three-month follow-up.

**Table 4:** Distribution of VAS score among the study participants (n = 110) VAS: visual analog scale.

VAS score	Frequency (%)		
	At postoperative period	After 1 month	After 3 months
0 (no pain)	0 (0)	0 (0)	0 (0)
1-3 (mild)	0 (0)	0 (0)	0 (0)
4-6 (moderate)	3 (2.4)	73 (66.4)	110 (100)
7-9 (severe)	107 (97.6)	37 (33.6)	0 (0)
10 (worst pain)	0 (0)	0 (0)	0 (0)

There was a statistically significant difference in the reduction of ODI score between the postoperative period and the one-month and three-month follow-up periods. As shown in Table 5, there was also a statistically significant difference in the reduction of VAS score after the postoperative period and at one and three months of follow-up. The ANOVA test revealed that these differences were statistically significant.

**Table 5:** Comparison of ODI and VAS scores among the study participants (n = 110) ODI: Oswestry Disability Index; VAS: visual analog scale.

Score		Mean + SD	ANOVA test (p-value)
Oswestry Disability Index score	Postoperative	17.90 + 4.4	0.001
	After 1 month	10.95 + 5.14	
	After 3 months	3.97 + 1.5	
Visual analog scale	Postoperative	7.45 + 0.5	0.0247
	After 1 month	6.14 + 0.71	
	After 3 months	5.30 + 1.21	

## Discussion

A total of 110 patients with dural tears were enrolled in this study. The mean age of the samples was 62.81 + 10.49 years. In a study by Ulrich et al., the mean age was 73 years [16]. Xiao et al. found that the mean age of participants in their study was 41.42 ± 12.68 years [17]. In a study conducted by Nandyala et al., the average age of the patients was 51 years [18]. The number of male participants in this study was markedly higher than the number of female participants. Similar findings were observed in a study by Tosun et al. [19], whereas in a study by Xiao et al. [17], the preponderance of females was higher than males.

The mean body mass index (BMI) of participants in this study was 23.77 kg/m2. Among the patients, 15.5% had the presence of hypertension, and 14.5% had diabetes mellitus. In a study by Ulrich et al., the mean BMI of participants was 26.1 kg/m2, 15.5% of the patients had hypertension, and 14.5% of patients had diabetes mellitus [16]. The participants in a study by Steurer et al. had a mean BMI of 26.3 kg/m2 [20].

In the present study, 16.4% of the participants had a smoking history, 8.2% had a history of tobacco chewing, and 8.2% had a drinking history. Of the total number of cases, 27 had a history of spinal surgery. A study conducted by Fokter et al. revealed that 35.6% of participants were dependent on alcohol [21]. Similarly, a study conducted by Hansraj et al. revealed that more than 40% of participants had a history of spine surgery [22].

In the majority of cases, urinary retention was reported as the primary symptom, followed by CSF leakage and postural headache. One case developed multiple myelodysplastic syndromes, and the other case developed pulmonary aspiration embolism. In two patients, surgical site infections were identified. In a research conducted by Cammisa et al. [12] and Saxler et al. [23], the most frequent complications associated with durotomies were headache, wound infection, meningeal pseudo-cyst, or dural cutaneous CSF fistulas resulting in meningitis and arachnoiditis. In a research conducted by Kalevski et al. [24], the majority of postoperative complications were CSF leakage following dural tears, which can pose potentially serious problems such as CSF fistula formation, pseudomeningocele, meningitis, arachnoiditis, and epidural abscess.

In the current study, a statistically significant difference in ODI scores was discovered postoperatively and at one-month and three-month follow-ups. Additionally, a statistically significant difference between VAS scores following surgical care and at follow-ups of one month and three months was discovered. The research by Grannum et al. [25] produced similar results, showing patients with dural tears appeared to have better improvements in outcome measures among the VAS and ODI scores. In their study, Desai et al. reported that there was a significant difference in ODI score during the follow-up period but no significant mean difference between the durotomy and no durotomy groups [26].

Over the past 40 years, there has not been much of a shift in the fundamentals of treating dural tears. An initial repair, Trendelenburg testing, watertight multilayer closure of the muscle and fascia, and four to seven days of bed rest in the supine position are all part of this procedure. The standard of care has been bed rest for at least 48 hours after repair, with or without the installation of a drain. Spinal headaches and CSF fistulas are less likely to occur due to the muscle-splitting technique and reduced potential (dead) space produced during minimally invasive spine surgery (MISS). The surgical procedure involves using either DuraSeal or Tisseel after performing the initial repair and/or DuraGen. Postoperative care required early mobilization less than 48 hours after surgery without the use of a drain, despite the fact that the amount of time spent in bed varied [27].

Ishaver et al.'s study was the first to directly compare the effects of early versus delayed mobilization and various repair modalities, albeit retrospectively [28]. The results clearly demonstrate that mandatory bed rest after a tear does not provide any benefit. Although not statistically significant, long-term bed rest would, in fact, appear to be harmful and increase the likelihood of long-term CSF leakage complications. We can also see that, in cases of minimally invasive spine surgery, a good, waterproof, layered closure is adequate without direct dural replacement.

The utilization of drains has been the subject of considerable debate. However, according to Cammisa et al., the use of a drain is contingent upon the nature of the procedure, the extent of the tear, the quality of the tissue, and the degree of repair of the tear [12].

Six consecutive patients with surgical CSF fluid leakage were documented by Patel et al. as receiving percutaneous fibrin sealant treatment [29]. The adhesion of the fibrin was verified by CT imaging after the spinal fluid was extracted under the direction of the machine and concurrently injected with a calcium chloride and thrombin solution and a cryoprecipitate solution. Three patients saw a rapid improvement in their symptoms; two required repeat surgery due to the recurrence of severe symptoms, and one patient's leak was not adequately covered with fibrin glue. There is currently no real agreement on how to handle inadvertent durotomies; each surgeon will approach the case differently. Primary repair techniques include the use of sutures, glue, muscle, fat, or fascial grafts, as well as blood or fibrin patches.

Limitations

Due to its hospital setting, this research cannot be extrapolated to the general public. However, we only followed up with patients for a total of three months, which is insufficient for determining the long-term outcome. Due to the limited duration of the follow-up period, the true extent of postoperative complications may be underestimated. The fact that different people performed the surgeries introduces the potential for procedural variability, and the lack of a validated clinical assessment score is another limitation.

## Conclusions

Among 110 patients, 32 had postoperative complications. Based on the ODI score, mild disability was seen among 32.7% of the study samples at three months of follow-up. Based on the VAS score, moderate pain was seen among all the study samples at three months of follow-up. There was a statistically significant difference in the reduction of ODI score and VAS score postoperatively and at follow-up of one month and three months (p-value of 0.001 and 0.0247, respectively) by ANOVA test.
